# Differential Effects of Red Meat/Refined Grain Diet and Dairy/Chicken/Nuts/Whole Grain Diet on Glucose, Insulin and Triglyceride in a Randomized Crossover Study

**DOI:** 10.3390/nu8110687

**Published:** 2016-10-30

**Authors:** Yoona Kim, Jennifer B. Keogh, Peter M. Clifton

**Affiliations:** School of Pharmacy and Medical Sciences, University of South Australia, Adelaide, SA 5000, Australia; yoona.kim@mymail.unisa.edu.au (Y.K.); jennifer.keogh@unisa.edu.au (J.B.K.)

**Keywords:** red meat/refined grain diet, dairy/chicken/nuts/whole grain diet, meal tolerance test, postprandial glucose, postprandial insulin

## Abstract

Epidemiological studies suggest that a diet high in processed meat, with a high glycemic index is associated with an increased risk of type 2 diabetes. It is not clear if this is due to altered insulin sensitivity or an enhanced postprandial glucose. We aimed to compare the acute metabolic response of two different types of meals after ingestion of the matching diet for four weeks. The study was a randomized, crossover acute meal study. Volunteers consumed either a red meat/refined grain meal or a dairy/chicken/nuts/wholegrain meal after four weeks of the matching diet. After a three-week washout period and four weeks of the alternate diet, they consumed the matching meal. The diets differed with respect to both protein and carbohydrate sources. Blood samples were taken for 180 min for the measurement of glucose, insulin, C-peptide and triglyceride. Fifty-one participants (age: 35.1 ± 15.6 years; body mass index: 27.7 ± 6.9 kg/m^2^, 17 with normal and 34 with impaired glucose tolerance) completed two meal tests. The area under the curve (*p* < 0.001) and incremental area under the curve (*p* = 0.001) for insulin was significantly higher after the red meat/refined grain diet than after the dairy/chicken/nuts/whole grain diet. There was an interaction between meal and glucose tolerance group (*p* < 0.05) in the area under the curve (AUC) and the incremental area under the curve (iAUC) of glucose; the red meat/refined grain diet increased glucose relative to the dairy/chicken/nuts/whole grain diet only in the normal group (+2.5 mmol/L/3 h). The red meat/refined grain diet increased glucose and insulin responses compared with the dairy/chicken/nuts/whole grain diet. This meal pattern would increase pancreatic stress long term and may account for the increased risk of type 2 diabetes with this diet.

## 1. Introduction

The prevalence of diabetes worldwide is rapidly rising from 382 million people in 2013 to an estimated 592 million by 2035 [1]. Diabetes prevention strategies are vital to reducing the health expenditure on diabetes and its complications [2,3]. Insulin resistance plays a key role in incidence of type 2 diabetes (T2DM) [4]. Weight loss, along with regular exercise, can reduce insulin resistance [5,6]. Changing dietary patterns without caloric restriction has been little investigated despite the positive epidemiology [7,8].

Prospective studies showed that a Mediterranean dietary pattern, rich in whole grains, nuts, fresh vegetables, fruit, moderate in dairy products and fish or poultry, and low in red and processed meet, refined grains and sugar-sweetened foods and beverages, was strongly associated with a decreased risk for T2DM ranging from 12% to 85% [9]. Fruits and vegetables alone have not been strongly linked to diabetes and there are no positive intervention studies [10,11]. 

Meta-analyses of prospective studies showed that processed meat intake of 50 g/day was associated with an increased risk of T2DM, ranging from 19% to 51% [12,13,14]. A Western dietary pattern high in red and processed meat and refined grains showed a positive association with the risk of T2DM in men [15]. There are no intervention studies on the effects of processed meat on insulin resistance or glucose homeostasis and this study directly addresses this question. The PREDIMED trial showed that a Mediterranean diet supplemented with mixed nuts reduced the risk of T2DM by 18% in the absence of weight change compared with a control diet after 4.1 years of follow-up [16]. 

Although the association between a diet high in red and processed meat and refined grains and an increased risk of T2DM is clear, it unknown if this is attributable to decreased insulin sensitivity or enhanced postprandial glucose responses or due entirely to confounding. These questions cannot be addressed without an intervention study. Therefore, this study aimed to determine the acute effects of two different types of meals on postprandial glucose, insulin, C-peptide and triglyceride after consumption of a matching diet for four weeks in order to elucidate mechanisms for changes in insulin sensitivity during the dairy/chicken/nuts/wholegrain diet.

We hypothesized that a meal consisting of white bread, leg ham and orange juice would increase postprandial glycemia and insulinemia compared with a meal consisting of whole grain bread, chicken, milk, yogurt and nuts matched for total energy. 

## 2. Methods

### 2.1. Participants

**Figure 1:**
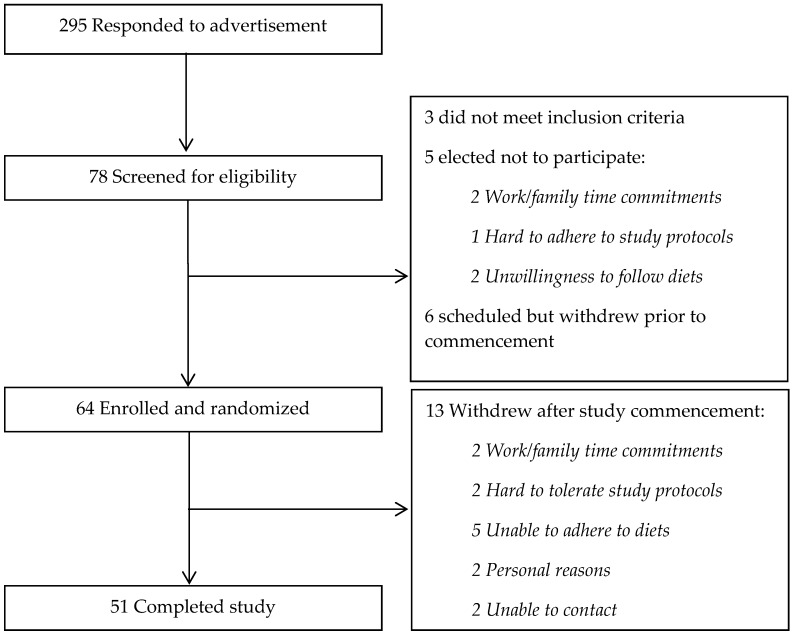
A participant flow chart.

Fifty-one participants were enrolled in this acute meal study. Eligible participants were adults aged over 18 years (body mass index (BMI), 18–45 kg/m^2^) without type 2 diabetes. Exclusion criteria were medication or supplements that could affect glucose metabolism, a history of metabolic illness including liver or kidney disease and allergy or intolerance to dairy, nuts or other components of the diet. Women who were pregnant or breastfeeding or likely to be pregnant and people who experienced weight gain or weight loss over the last three months were also excluded. A recruiting agency and public advertisement were used for the recruitment. An initial screening questionnaire was conducted via email or telephone to identify suitable participants. People who passed the screening criteria visited the Sansom Institute for Health Research Clinical Trial facility at the University of South Australia, in the morning after an overnight fast. A 75 g oral glucose tolerance test (OGTT) was undertaken to exclude people with frank diabetes. This study was approved by the University of South Australia Human Research Ethics committee and registered with the Australian New Zealand Clinical Trials Registry (ACTRN12614000519651). Written, informed consent was obtained. Recruitment started in June 2014 and ended in September 2015. Participant recruitment and completion is shown in Figure 1. All participants who completed both chronic dietary phases had meal tolerance tests (MTTs) analyzed. Those with only one MTT were not analyzed.

### 2.2. Dietary Intervention

This study was part of a randomized crossover trial with two different dietary patterns which will be published separately. The effect of the chronic diets on insulin sensitivity was assessed with a low dose infusion of glucose and insulin. Participants followed either a diet high in red meat and processed meat and refined grains or a diet high in whole grains, nuts, and dairy products and no red meat for four weeks and then were crossed-over to the other dietary pattern. Food was not given to participants but an AU$ 50 and an AU$ 80 voucher were provided for the purchase of nuts and red meat, respectively, at the end of each diet period. Participants were specifically instructed regarding what to eat for each four-week period by providing eight different dietary guidelines in accordance with BMI and gender. The dietary guidelines included daily food selection goals, samples of a meal plan and recipes, cooking methods and a standard serving size guide. A kitchen scale was provided to participants. Participants were free-living but were asked not to change their lifestyle and to maintain body weight. Participants were encouraged to contact investigators whenever they had inquiries via telephone or email. The two diets were intended to be isocaloric. Daily checklists and three-day weighed food diaries recorded fortnightly including cooking technique, food brand names, quantity and leftovers, were collected to ensure dietary compliance. Food-intake data (2 × 3-day weighed food diaries) were analyzed with FoodWorks Professional Edition 8.0 (Xyris Software, Brisbane, Australia). The recommended menu for the two different diets is shown in Table 1. 

There was on average a three-week washout period on their usual diet in between the two four-week weight-stable diet periods. Randomization was undertaken using an online random number generator (www.randomization.com) by a researcher not involved in the study. The investigator who carried out data analysis and staff who measured blood samples were unaware of the diet order of participants. The MTT was conducted in the morning after an overnight fast at the end of each four-week diet period. The meal order in MTT was based on the diet that volunteers had followed for four weeks, which was randomized. Two different kinds of sandwiches were created in accordance with the key food profiles of two different diets. Food was not provided in the four-week diet intervention. The ham sandwich, representing a diet high in red meat and processed meat and refined grains was comprised of: 80 g white bread, 105 g leg ham, 20 g iceberg lettuce, 10 g tomato slices, 3 g onions, 3 g pickles and 250 mL 25% orange juice. The chicken sandwich, representing a diet high in whole grains, nuts and dairy products and no red and processed meat consisted of: 80 g wholegrain bread, 50 g chicken breast, 20 g iceberg lettuce, 10 g tomato slices, 100 g natural Greek yogurt, 200 g fat reduced milk, 7 g cashew nuts and 7 g peanuts. Participants were advised to consume the meal within 10 min. The food composition of each meal is summarized in Table 2. The two test meals were designed to be enriched in red meat or enriched in dairy but contained the same energy. Other macronutrients differed. The glycemic load of the dairy/chicken/nuts/wholegrain meal was 58% of the red meat/refined grain meal.

### 2.3. Clinical Measurements

An intravenous catheter (BD Nexiva catheters; 20GA 1.25IN 1.1 × 32 mm, Becton Dickinson and Co., Franklin Lakes, NJ, USA) was placed in a large antecubital vein. Blood samples were taken at −5, 0, 10, 20, 30, 40, 50, 60, 75, 90, 120, 150, and 180 min into both a tube with a sodium fluoride EDTA (ethylenediaminetetracetic acid) and a tube with no additives. The tube with no additives for serum insulin, C-peptide and triglyceride (TG) was stood upright at room temperature for 30 min, and then it was placed on ice, whereas the sodium fluoride EDTA tube for plasma glucose was placed straight on ice until centrifugation and processing. Blood samples were centrifuged at 4000 rpm at 4 °C for 10 min (Universal 32R, Hettich Zentrifugen, Tuttlingen, Germany). Plasma and serum aliquots were stored at −80 °C until analysis. The measurements of plasma glucose and serum TG were made using an automated spectrophotometric analyzer (Konelab 20XTi, ThermoFisher Scientific, Waltham, MA, USA). Serum insulin and C-peptide analyses were conducted by commercial ELISA kits (Alpha Diagnostic, San Antonio, TX, USA, Kit # 0030N for insulin, Kit # 0040 for C-peptide). 

### 2.4. Analysis

Statistical analysis was performed using SPSS V22 (IBM, Chicago, IL, USA). 

A sample size of 50 overweight and obese participants without diabetes was required to have 80% power to detect a 20% change (alpha level = 0.05) in insulin sensitivity with 80% power as assessed by LDIGIT (low dose insulin and glucose infusion test) [17]. No specific calculation was made for the MTT.

The area under the curve (AUC) and incremental AUC (iAUC) for glucose, insulin, C-peptide and TG were calculated using the trapezoidal method. The Shapiro-Wilk test, Q-Q plots, and histograms were used to test for the normality of distribution. Non-normally distributed data such as fasting glucose, insulin AUC, insulin iAUC, C-peptide iAUC and TG AUC were log transformed. Differences between two meals were assessed by repeated-measures ANOVA. A Wilcoxon signed rank nonparametric test was used as fasting insulin was still skewed after log transformation. TG iAUC was also analyzed by a Wilcoxon signed rank nonparametric test. Data for glucose AUC, glucose iAUC and C-peptide AUC are presented as the mean ± standard deviation (SD). Data for skewed variables such as fasting glucose, fasting insulin, insulin AUC, insulin iAUC, C-peptide iAUC, TG AUC and TG iAUC are expressed as medians and interquartile ranges. Chi-square tests and unpaired *t* tests were performed to compare participants’ baseline characteristics by randomization order. Statistical significance was defined as *p* < 0.05. 

## 3. Results

Fifty-one participants (15 men and 36 women) completed the study (Figure 1). A total of 13 participants withdrew from the study after study commencement. Two participants withdrew on the day of the blood collection after first four-week diet period due to the fear of blood collection. Five participants withdrew from the diet within five days of the first diet commencement: three could not adhere to red meat/refined grain diet and two could not comply with dairy/chicken/nuts/wholegrain diet. Four participants were enrolled but withdrew due to work and family time commitments, and for personal reasons. Two participants could not be contacted. Baseline characteristics of all participants and baseline characteristics of participants by sequence order is shown in Table 3. This was a small difference in 2 h glucose between randomization order groups.

Analyses of six-day weighed food diaries showed that compliance with the protocol was satisfactory. Total energy intake did not differ between two diets. Reported dietary intake is shown in Table 4 and Table 5. Overall, intakes of key foods assessed by food diaries were similar to the recommended quantities of key foods. Body weight was measured at baseline, week two and week four. Mean body weight was almost unchanged throughout the intervention period.

**Figure 2:**
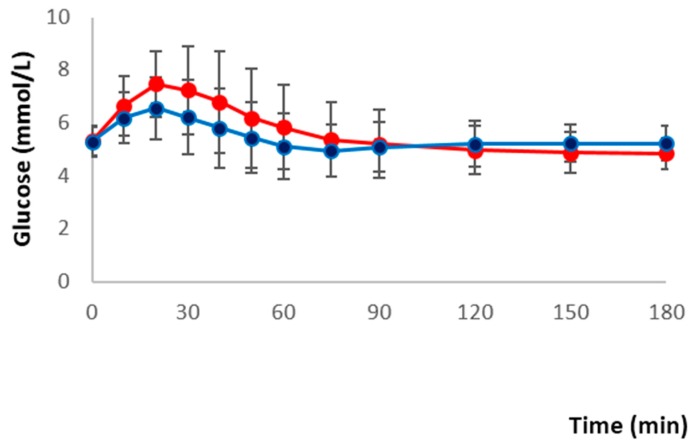
Mean ± standard deviation (SD) changes in postprandial glucose concentrations after two diets (*n* = 51). A repeated-measures ANOVA showed significance for the treatment (*p* = 0.003) and time-by treatment interaction (*p* < 0.001; *n* = 51). 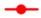 red meat/refined grain diet; 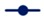 dairy/chicken/nuts/whole grain diet.

**Figure 3:**
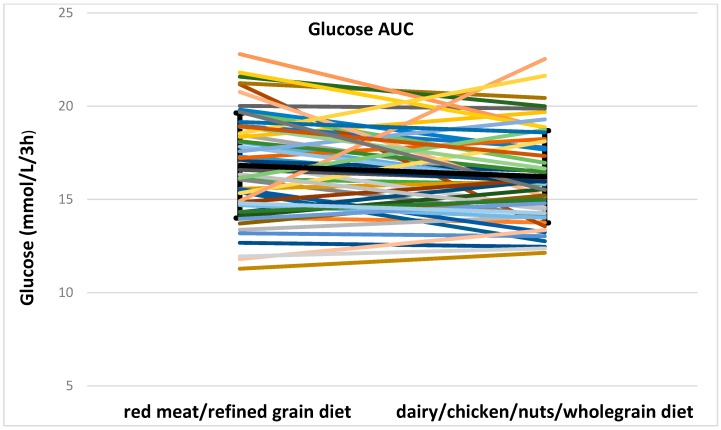

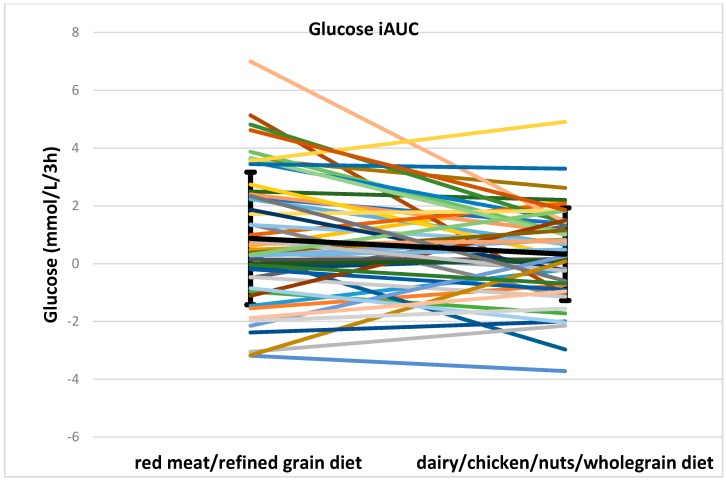
Individual data for glucose AUC and iAUC. The bold black line shows the mean and SD. AUC, area under the curve; iAUC, incremental area under the curve.

Fasting glucose concentrations between two meals did not differ (median, interquartile range 5.3, 0.9 mmol/L for red meat/refined grain diet vs. 5.3, 0.6 mmol/L for dairy/chicken/nuts/whole grain diet). Total glucose AUC was not different between two meals (16.8 ± 2.8 mmol/L/3 h for red meat/refined grain diet vs. 16.2 ± 2.5 mmol/L/3 h for dairy/chicken/nuts/whole grain diet). The iAUC for glucose significantly differed between two meals (0.9 ± 2.3 mmol/L/3 h for red meat/refined grain diet vs. 0.3 ± 1.6 mmol/L/3 h for dairy/chicken/nuts/wholegrain diet, *p* < 0.05). There was a group by meal effect (*p* < 0.05 for total glucose AUC and *p* = 0.016 for total glucose iAUC) so glycemic control groups were analyzed separately. The group with normoglycemia (*n* = 17) had a significant diet effect for total glucose AUC (*p* = 0.017) and total glucose iAUC (*p* = 0.024) while in the group with impaired fasting glucose (IFG) or impaired glucose tolerance (IGT) diet (*n* = 34) was not insignificantly different. Postprandial glucose concentrations after two meals are shown in Figure 2. Individual data for glucose AUC and iAUC is shown in Figure 3.

**Figure 4:**
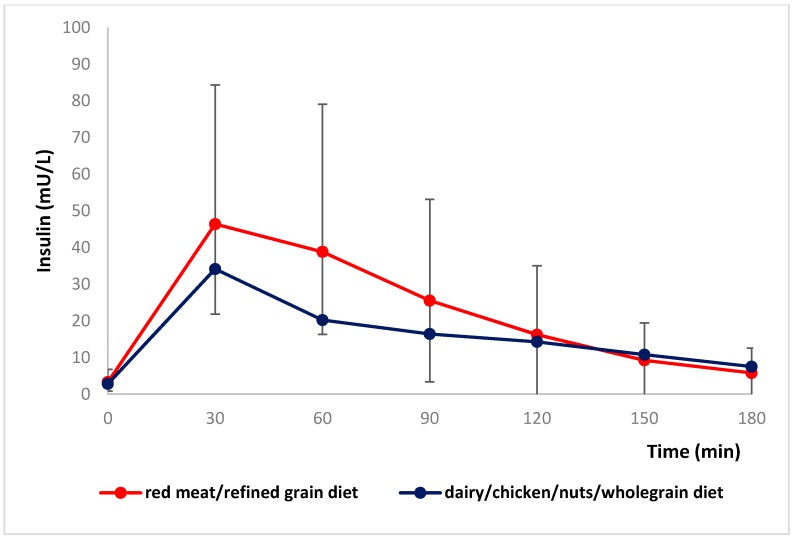
Mean ± SD changes in postprandial insulin concentrations after two diets (*n* = 51). A repeated-measures ANOVA of log transformed data showed significance for the treatment (*p* = 0.004) and time-by-treatment interaction (*p* < 0.001; *n* = 51).

**Figure 5:**
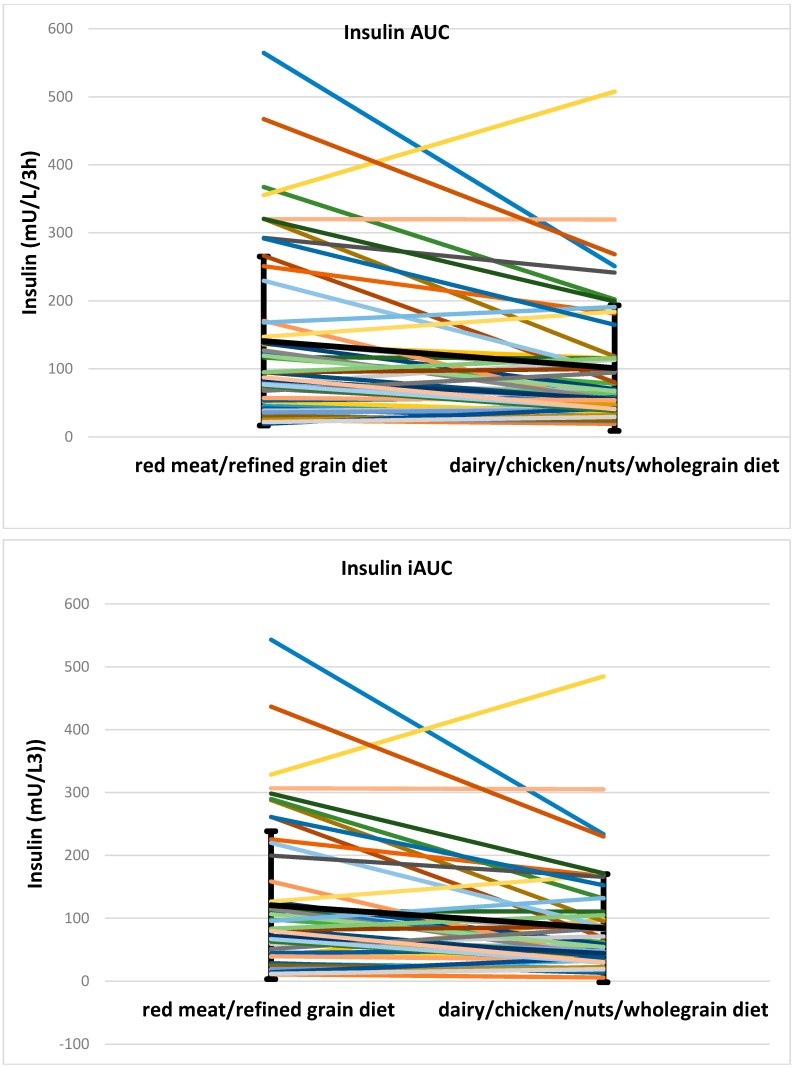
Individual data for insulin AUC and iAUC. The bold black line shows the mean and SD. AUC, area under the curve; iAUC, incremental area under the curve.

**Figure 6:**
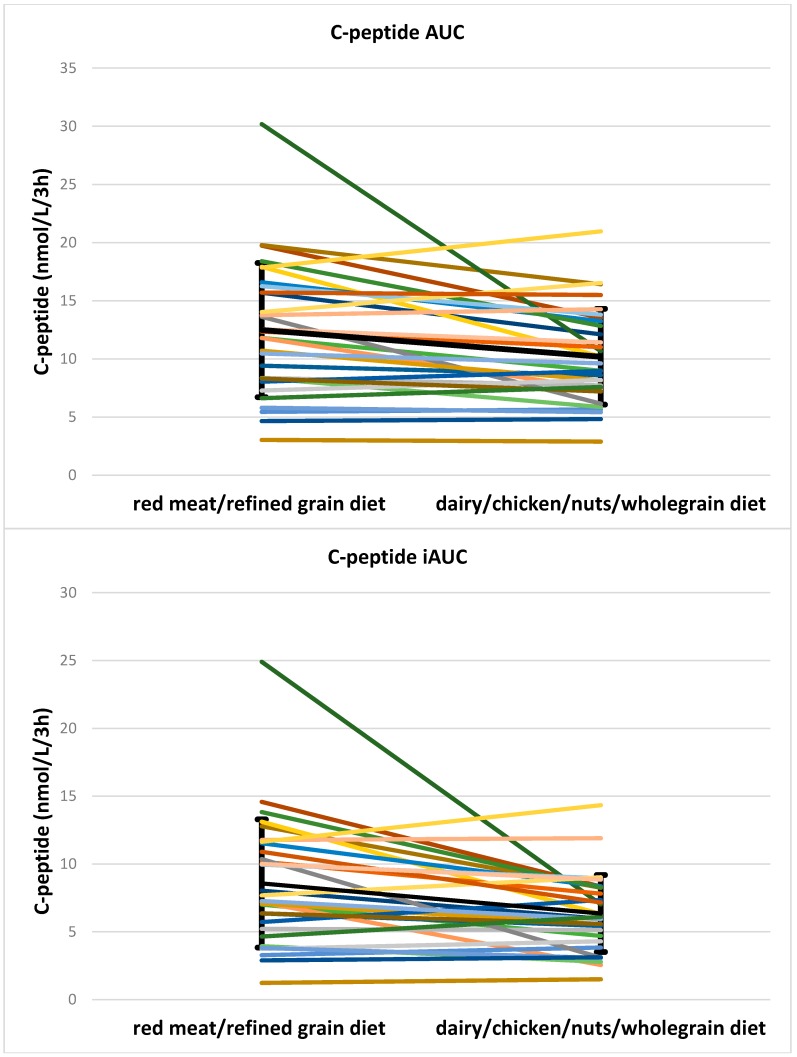
Individual data for C-peptide AUC and iAUC. The bold black line shows the mean and SD. AUC, area under the curve; iAUC, incremental area under the curve.

There was a small difference in fasting insulin, which was higher after the dairy/chicken /nuts/wholegrain diet vs. the red meat/refined grain diet. However, insulin AUC after red meat/refined grain diet was significantly higher than for the dairy/chicken/nuts/whole grain diet (94.4, 117 mU/L/3 h for red meat/refined grain diet vs. 65.9, 74.3 mU/L/3 h for dairy/chicken/nuts/wholegrain diet, *p* < 0.001). The insulin iAUC was significantly higher after the red meat/refined grain diet (79.7, 119.2 mU/L/3 h for red meat/refined grain diet vs. 54.3, 71.9 mU/L/3 h for dairy/chicken/nuts/whole grain diet, *p* = 0.001) than after dairy/chicken/nuts/wholegrain diet. Postprandial insulin concentrations after the two diets are shown in Figure 4. Individual data for insulin AUC and iAUC is shown in Figure 5. Both the C-peptide AUC (12.5 ± 5.8 nmol/L/3 h for red meat/refined grain diet vs. 10.2 ± 4.1 nmol/L/3 h for dairy/chicken/nuts/wholegrain diet, *p* = 0.006) and iAUC (8.6, 6.5 nmol/L/3 h for red meat/refined grain diet vs. 6.4, 4.1 nmol/L/3 h for dairy/chicken/nuts/wholegrain diet, *p* = 0.002) were higher after the red meat/refined grain diet compared to the dairy/chicken/nuts/wholegrain diet. There was no interaction with glycemic group. Individual data for C-peptide AUC and iAUC is shown in Figure 6.

**Figure 7:**
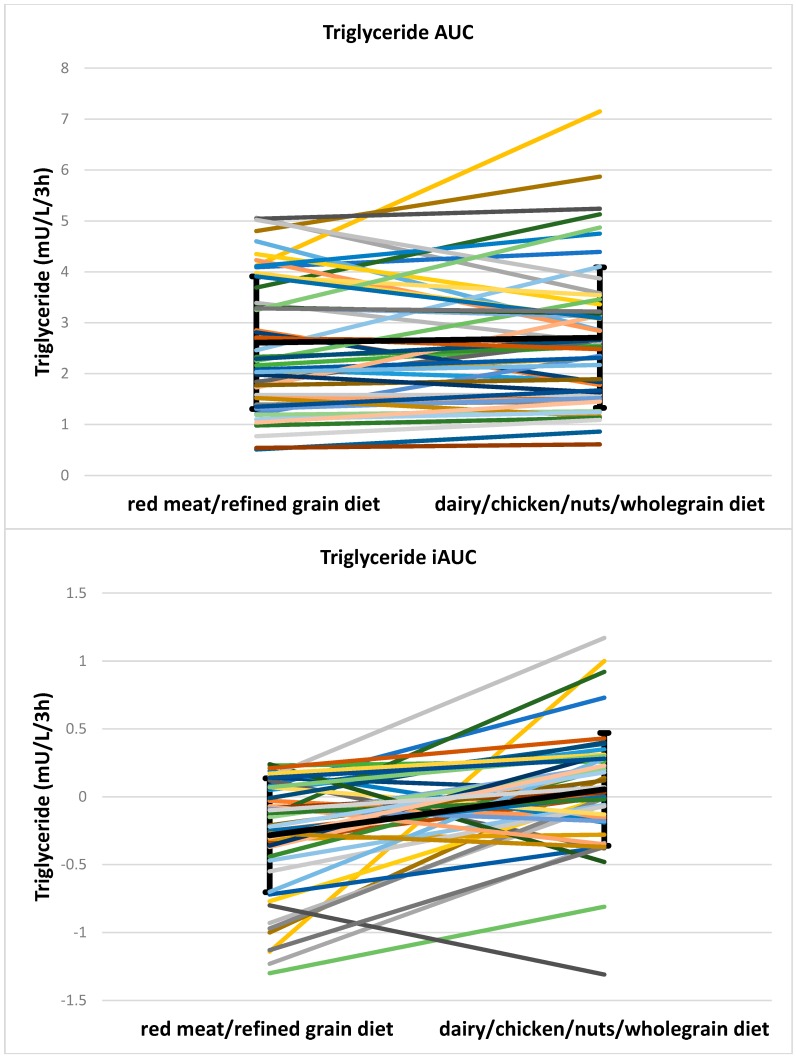
Individual data for triglyceride AUC and iAUC. The bold black line shows the mean and SD. AUC, area under the curve; iAUC, incremental area under the curve.

TG AUC did not differ between two meals (2.3, 2.4 mU/L/3 h for red meat/refined grain diet vs. 2.6, 1.8 mU/L/3 h for dairy/chicken/nuts/wholegrain diet, *p* = 0.172) but there was a small significant difference in TG iAUC (−0.2, 0.5 mU/L/3 h for red meat/refined grain diet vs. 0.0, 0.4 mU/L/3 h for dairy/chicken/nuts/wholegrain diet, *p* < 0.001). Individual data for triglyceride AUC and iAUC is shown in Figure 7. Differences in glucose, insulin, C-peptide and TG are presented in Table 6. 

## 4. Discussion

In this study, a red meat/refined grain meal resulted in significantly higher postprandial insulin secretion than a dairy/chicken/nuts/wholegrain meal after a similar chronic dietary pattern but did not alter postprandial glucose concentrations. The 27% increase in glycemic load in the red meat/refined grain meal only partially explains the 40%–50% rise in insulin. Therefore, the significantly increased insulin concentrations could be also partly attributable to the four-week red meat/refined grain diet high in refined grains, red and processed meat promoting insulin resistance. The population tested was very diverse from lean insulin sensitive to obese insulin resistant subjects, some of whom had impaired glucose tolerance, so we believe the findings apply to the whole population. Our finding suggests that meals varying moderately in carbohydrate content and glycemic index (GI) do not influence total blood glucose to any major degree. 

This finding is consistent with the small study of 25 overweight subjects where there was no difference in mean 24-h or daytime glucose concentrations on diets differing in carbohydrate and glycemic index (GI) [18]. It is interesting in this data set that an increase in glucose was seen only in the normoglycemic group who would be expected to mount a good insulin response but insulin differences between meals were not related to glycemic control group. 

C-peptide only increased by 23% for total AUC on the red meat/refined grain diet while insulin increased by 46% compared with the diary/chicken/nuts/wholegrain diet, suggesting that the large difference in insulin levels was due to lowered insulin clearance rather than increased insulin release with the red meat/refined grain diet. This suggests the red meat/refined grain diet for four weeks had reduced insulin sensitivity and one of the early responses with changes in insulin sensitivity is alteration in hepatic insulin clearance rates. Therefore, only half of the increase in insulin was attributable to increased glycemic load and the other half was due to insulin resistance. Several studies showed that decreased insulin sensitivity was associated with decreased insulin clearance [19,20,21], and reduced insulin clearance correlated with risk of incidence of T2DM [22]. The Insulin Resistance Atherosclerosis Study 5-year follow-up showed a negative correlation between plasma plasminogen activator inhibitor (PAI)-1 concentrations and insulin clearance [23]. Dietary factors such as low GI foods might reduce PAI-1 activity [24,25].

The dairy/chicken/nuts/wholegrain diet contained a reasonable amount of nuts. Dietary fiber in nuts slows gastric emptying and subsequent glucose absorption [26]. Acute nut ingestion usually blunts the postprandial glucose response to carbohydrate foods due to their high fat content as well [27,28]. A pooled analysis of 25 intervention studies showed that the nut consumption of 67 g/day reduced fasting triglyceride concentrations by 10.2% only in people with hypertriglyceridemia but no overall triglyceride-lowering effect of nuts was seen [29]. The dairy/chicken/nuts/wholegrain meal in this study contained 9 g more fat and this was reflected in slightly higher postprandial triglyceride levels.

Studies investigating effects of isoenergetic diets differing in dietary protein and cereal fiber content on insulin sensitivity indicated that a high protein diet was associated with increased insulin resistance compared with a high cereal fiber diet [30,31]. This emphasizes the importance of high quality carbohydrate rich in fiber as observed in our diet and meal of dairy/chicken/nuts/wholegrain. 

In line with our finding, Leinonen et al. [32] found that whole kernel rye bread lowered the postprandial insulin response compared with wheat bread, but there was no difference in glucose response between the two breads in healthy subjects. Whey protein has been shown to have an insulinotropic effect compared with non-dairy proteins [33] in some studies but not all [34]. Whey protein consumed in amounts of 20 g/day before or after a carbohydrate meal stimulated insulin, glucagon-like peptide-1 (GLP-1) and gastric inhibitory polypeptide (GIP) and delayed gastric emptying resulting in decreased postprandial glycaemia in people with diet-controlled T2DM [35,36]. It appears that 2.8 g whey in milk and yogurt of our dairy/chicken/nuts/wholegrain meal has no effect on postprandial glycemia and insulin secretion and that meat and dairy protein probably have equivalent effects on insulin release. As there are inconsistent effects of dairy consumption on insulin sensitivity [37,38] and few dairy interventions examining acute effects on glucose and insulin [34] exist, the possible explanation for overall effects observed in the dairy/chicken/nuts/wholegrain meal of this present study are not due to dairy but other meal components. Alteration in gut microbiota may play a role in the beneficial effects seen with the dairy/chicken/nuts/wholegrain diet [39].

A limitation of this study was that the macronutrient compositions were not matched and total energy content was slightly different between two meals leading to very slight differences in triglyceride incremental area. A further limitation was that we have no baseline measurements to show that there was no carry-over of the previous diet. However, diet order had no effect on the results, suggesting there was no significant carry-over and the results observed are valid. The study design also assumed that all prognostic factors other than the treatment remained constant across treatment conditions. Randomization might not work to equalize the populations as the sample size was small and there was a small difference in 2-h glucose between the two groups. Even though the compliance was good, self-reported food intake [40] and meal preparation outside the clinical research facility during the intervention periods also could be a potential limitation. 

## 5. Conclusions

In conclusion, a red meat/refined grain meal following four weeks of a similar red meat/refined grain diet elevated insulin and incremental glucose responses in comparison with a dairy/chicken/nuts/whole grain meal, which is consistent with the increased insulin resistance which we observed in relatively insulin-resistant adults in the main study as well as the carbohydrate quality and quantity differences between the meals. The adverse insulin response to the red meat/refined grain meal relative to the dairy/chicken/nuts/wholegrain meal indicates possible mechanisms through which these diets might affect risk of type 2 diabetes. Further work needs to be done on individual components of the meal to define which is the most important factor. The population in this study consisted of 63% Caucasian, 28% Asian and 9% Latin American subjects with over 50% aged <40 years. Future studies should attempt to replicate the results in a population including middle-aged people from the Middle East and the Pacific area.

## Figures and Tables

**Table 1:** The recommended menus for each of the diets.

Red Meat/Refined Grain Diet		Dairy/Chicken/Nuts/Whole Grain Diet	
Food	Daily Goal	Food	Daily Goal
Red meat	200–300 g	Chicken Fish Cooked legumes	80–150 g 70–150 g 150–225 g (l cup–1.5 cups)
Processed meat	≥50 g	Nuts	60–90 g
Refined grains	4–6 serves	Whole grains	3–4 serves
Dairy products	minimal	Dairy products	4 serves
Potato	200–300 g		
Vegetables	1–2 serves	Vegetables	1–2 serves
Fruits	1–2 serves	Fruits	1–2 serves
Oil/spread	3–9 serves	Oil/spread	2–7 serves
Jam or marmalade	1 serve	Jam or marmalade	≤1 serve
Wine/Alcohol	Optional	Wine/Alcohol	Optional
Indulgence food	3–4 serves To replace refined grains or potato if desired		

Serving sizes were based on the guidelines of the Australian National Health and Medical Research Council.

**Table 2:** Nutrient composition of the test meals.

	Amount (g)	Energy (kJ)	CHO (g)	Food GI	Protein (g)	Fat (g)	Sat Fat (g)	Cholesterol (mg)	Sugar (g)	Dietary Fibre (g)	Sodium (mg)
**Red meat/refined grain meal **											
Bread, white,	80	848	39.2	70	6.4	1.52	0.24	0	2.32	2.16	320
25% orange juice	250	481	26.8	52	0.3	0	0	0	25.7	0.2	1
Lettuce, iceberg, raw	20	8	0.08		0.2	0.02	0	0	0.08	0.3	5.2
Tomato, raw	10	7.4	0.24		0.1	0.01	0	0	0.23	0.12	0.8
Ham, leg, lean & fat	105	804	1.5		20.7	11.6	4.7	73.5	0.1	0	1614
Onion, raw	3	3.81	0.14		0.05	0	0	0	0.14	0.06	0.3
Pickle	3	10.44	0.5		0.02	0.02	0	0	0.44	0.04	18.6
Total		**2162.65**	**68.46**	**60.4**	**27.77**	**13.17**	**4.94**	**73.5**	**29.01**	**2.88**	**1959.9**
**Dairy/chicken/nuts/wholegrain meal**											
Bread, wholegrain,	80	840	34.6	53	7.6	2.56	0.32	0	2.8	4.16	320
Nuts, cashews, raw	7	170.59	1.18	22	1.19	3.44	0.59	0	0.39	0.41	0.77
Nuts, peanuts, raw	7	166	1.12	14	1.82	3.43	0.49	0	0.28	0.63	1.26
Yoghurt, greek, natural	100	557	6.9	27	4.7	9.7	6.4	-	6.9	0	62
Chicken, breast, lean, raw	50	219	0		11.15	0.8	0.25	29.5	0	0	20.5
Milk, reduced fat	200	372	9.6	37	6.64	2.6	1.8	-	9.6	0	86
Lettuce, iceberg, raw	20	8	0.08		0.2	0.02	0		0.08	0.3	5.2
Tomato, raw	10	7.4	0.24		0.1	0.01	0		0.23	0.12	0.8
Total		**2339.99**	**53.72**	**44.9**	**33.4**	**22.56**	**9.85**	**29.5**	**20.28**	**5.62**	**496.53**

CHO, carbohydrate; GI, glycemic index.

**Table 3:** Baseline characteristics of a total of participants and participants by sequence order ^1^.

	All Participants	Participants Randomized to Red Meat/Refined Grain Diet First and Then Dairy/Chicken/Nuts/Whole Grain Diet (*n* = 27)	Participants Randomized to Dairy/Chicken/Nuts/Whole Grain Diet First and Then Red Meat/Refined Grain Diet (*n* = 24)	*p* Value
Sex (Male/Female)	15/36	9/18	6/18	0.5
Age (year)	35.1 ± 15.6	33 ± 12.7	37.4 ± 18.3	0.3
NGT (*n*)	17	10	7	0.6
IFG/IGT (*n*)	34	17	17	
Baseline fasting glucose (mmol/L)	5.5 ± 0.7	5.6 ± 0.7	5.4 ± 0.7	0.4
Baseline 2-h glucose (mmol/L)	7.3 ± 1.6	6.9 ± 1.3	7.8 ± 1.8	0.04 *
Baseline weight (kg)	79.4 ± 21.36	82.6 ± 21.4	75.8 ± 21.2	0.3
BMI (kg/m^2^)	27.7 ± 6.9	28.6 ± 7.0	26.7 ± 6.9	0.3
Baseline SBP (mmHg)	112.2 ± 10.7 ^2^	112.2 ± 12.4 ^3^	112.2 ± 8.4 ^4^	1
Baseline DBP (mmHg)	70.7 ± 9.7 ^2^	70.4 ± 9.7 ^3^	71.3 ± 9.9 ^4^	0.8
Total Fat Mass (kg)	29 ± 15.7	30.4 ± 15.6	27.4 ± 16.0	0.5
Total Lean Mass (kg)	46.6 ± 11.5	48.4 ± 12.3	44.6 ± 10.4	0.2
Total Fat Mass (%)	36.6 ± 12.6	37.3 ± 12.2	35.9 ± 13.3	0.7

*p* values were obtained by chi-square tests and unpaired t tests to compare participants’ characteristics by randomization order. Values are means ± SDs. NGT, normal glucose tolerance; IFG, impaired fasting glucose; IGT, impaired glucose tolerance; BMI, body mass index; SBP, systolic blood pressure; DBP, diastolic blood pressure. ^1^ *n* = 51; ^2^ *n* = 42; ^3^ *n* = 24; ^4^ *n* = 18.

**Table 4:** Reported dietary intake of key foods assessed by weighed food records ^1^.

Red Meat/Refined Grain Diet		Dairy/Chicken/Nuts/Wholegrain Diet	
Food	Average intake	Food	Average intake
Red meat	241.3 g	Chicken	105.5 g
		Fish	110.5 g
		Cooked legumes	154.3 g
Processed meat	55.8 g	Nuts	70 g
Refined grains	5.2 serves (320.4 g) *	Whole grains	3.9 serves (235.8 g) *
		Dairy products	3.8 serves (687.4 g)

These values are based on an average of two weighed food diaries per dietary period. ^1^ *n* = 51. * Weight (g) was calculated based on cooked for rice and pasta, and raw for cereal, oats and muesli.

**Table 5:** Reported dietary intake of nutrients assessed by weighed food records ^1^.

	Red Meat/Refined Grain Diet	Dairy/Chicken/Nuts/Whole Grain Diet	*p* Value
Energy (kJ)	8406 ± 1172	8382 ± 1078	0.76
Protein (g)	95, 13.7	97, 14	0.41
Total fat (g)	73 ± 18	87 ± 16	*p* < 0.001
Saturated fat (g)	26.2, 8.5	26, 9.3	0.93
Polyunsaturated fat (g)	8.7, 4.4	17.2, 6.6	*p* < 0.001
Monounsaturated fat (g)	31 ± 9	37 ± 7	*p* < 0.001
Cholesterol (mg)	219.4, 52	158, 67	*p* < 0.001
Carbohydrate (g)	218.3, 43	181,40	*p* < 0.001
Sugars (g)	61, 26	89, 26	*p* < 0.001
Starch (g)	151.3, 46	93, 43	*p* < 0.001
Alcohol (g)	0, 8.7	0, 1.8	*p* < 0.05
Dietary fibre (g)	17, 6.4	27,8	*p* < 0.001
Thiamine (mg)	1.5, 0.6	1.9, 0.5	*p* < 0.05
Riboflavin (mg)	1, 0.5	2.8, 0.7	*p* < 0.001
Niacin (mg)	46.1, 12	44, 9.4	0.12
Vitamin C (mg)	90.5, 50	54, 40	*p* < 0.001
Total folate (µg)	236, 157	386, 131	*p* < 0.001
Sodium (mg)	2092, 918	1588, 523	*p* < 0.001
Potassium (mg)	2886 ± 531	3441 ± 450	*p* < 0.001
Magnesium (mg)	229, 51	518, 138	*p* < 0.001
Calcium (mg)	245, 116	1418, 253	*p* < 0.001
Iron (mg)	11.5, 3	11.5, 4	0.7
Zinc (mg)	13.6, 3.8	12, 2.6	*p* < 0.001
Protein (% energy)	19.2, 3	19.6, 3	0.4
Fat (% energy)	32 ± 5	39 ± 5	*p* < 0.001
Carbohydrate (% energy)	44 ± 6	37 ± 5	*p* < 0.001
Fibre (% energy)	1.7, 0.4	2.7, 0.7	*p* < 0.001

These values are based on an average of two weighed food diaries per dietary period. ^1^ *n* = 51. Normally distributed values are presented as means ± SDs and *p* values were determined by paired *t* tests. Non–normally distributed variables (shown as medians and interquartile ranges) were log transformed and *p* values were determined by paired *t* tests for protein, saturated fat, polyunsaturated fat, cholesterol, carbohydrate, sugars, starch, dietary fiber, sodium, calcium, zinc and % energy from fiber, and Wilcoxon signed rank nonparametric tests were performed for alcohol, thiamin, riboflavin, niacin, vitamin C, folate, magnesium, iron and % energy from protein.

**Table 6:** Effects of meals on glucose, insulin and TG measured at the end of each diet period.

	All (*n* = 51)				Group with NGT (*n* = 17)			Group with IFG or IGT (*n* = 34)		
	Red Meat/Refined Grain Diet	Dairy/Chicken/Nuts/Wholegrain Diet	*p* Value	*p* Group by Meal	Red Meat/Refined Grain Diet	Dairy/Chicken/Nuts/Wholegrain Diet	*p* Value	Red Meat/Refined Grain Diet	Dairy/Chicken/Nuts/Wholegrain Diet	*p* Value
Fasting glucose (mmol/L)	5.3, 0.9	5.3, 0.6	0.828	NS	5.2, 0.6	5.2, 0.8	0.59	5.4, 0.9	5.4, 0.6	0.99
Glucose AUC (mmol/L/3 h)	16.8 ± 2.8	16.2 ± 2.5	0.077	0.047	16.5 ± 2.9	14.9 ± 2.0	0.017	17 ± 2.8	16.9 ± 2.4	0.7
Glucose iAUC (mmol/L/3 h)	0.9 ± 2.3	0.3 ± 1.6	0.046	0.016	1.1 ± 2.9	−0.3 ± 1.7	0.024	0.8 ± 1.9	0.7 ± 1.5	0.7
Fasting insulin (mU/L)	2.2, 1.9	2.2, 1.2	0.023	NS	2.1, 1.1	2.1, 0.88	0.78	2.5, 2.1	2.3, 1.4	0.012
Insulin AUC (mU/L/3 h)	94.4, 117	65.9, 74.3	<0.001	NS	116.5, 169.5	65.9, 116.6	0.012	85.8, 129.4	62.1, 74.1	0.001
Insulin iAUC (mU/L/3 h)	79.7, 119.2	54.3, 71.9	0.001	NS	95.7, 120.7	54.3, 92.7	0.033	70.2, 134.7	50.8, 67.9	0.008
C-peptide AUC (nmol/L/3 h)	12.5 ± 5.8	10.2 ± 4.1	0.006	NS	10.9 ± 5.0	9.0 ± 4.2	0.075	13.3 ± 6.1	10.8 ± 4.1	0.03
C-peptide iAUC (nmol/L/3 h)	8.6, 6.5	6.4, 4.1	0.002	NS	6.7, 7.3	5.1, 4.6	0.112	7.9, 6.1	6.2, 3.1	0.014
TG AUC (mU/L/3 h)	2.3, 2.4	2.6, 1.8	0.172	0.013	2.0, 1.5	2.4, 2.1	0.008	2.8, 2.3	2.7, 1.7	0.78
TG iAUC (mU/L/3 h)	−0.2, 0.5	0.0, 0.4	<0.001	NS	−0.2, 0.8	0.2, 0.5	0.004	−0.2, 0.5	0.0, 0.4	0.001

Fasting glucose, insulin AUC, insulin iAUC, C-peptide iAUC and TG AUC were log transformed. *p* values for fasting insulin and TG iAUC were obtained from Wilcoxon signed rank nonparametric tests as fasting insulin and TG iAUC were not still normally distributed after log transformation. *p* values for fasting glucose, glucose AUC, glucose iAUC, insulin AUC, insulin iAUC, C-peptide AUC, C-peptide iAUC and TG AUC were examined by paired t tests. Values are expressed as medians and interquartile ranges except for glucose AUC, glucose iAUC and C-peptide AUC, which are presented as means ± SDs. Group 1 was comprised of participants with normal glucose tolerance (NGT) and group 2 was comprised of participants with impaired fasting glucose (IFG) and impaired glucose tolerance (IGT). AUC, area under the curve; iAUC, incremental area under the curve; TG, triglyceride. For AUC C-peptide *n* = 10 with NGT and 20 with IFG/IGT.
